# A biotroph sets the stage for a necrotroph to play: ‘*Candidatus* Phytoplasma solani’ infection of sugar beet facilitated *Macrophomina phaseolina* root rot

**DOI:** 10.3389/fmicb.2023.1164035

**Published:** 2023-04-20

**Authors:** Nataša Duduk, Ivana Vico, Andrea Kosovac, Jelena Stepanović, Živko Ćurčić, Nina Vučković, Emil Rekanović, Bojan Duduk

**Affiliations:** ^1^Faculty of Agriculture, University of Belgrade, Belgrade, Serbia; ^2^Institute of Pesticides and Environmental Protection, Belgrade, Serbia; ^3^Institute of Field and Vegetable Crops, Novi Sad, Serbia

**Keywords:** phytoplasma fungus complex, stolbur phytoplasma, RTD, rubbery taproot disease, *Reptalus quinquecostatus*, *Beta vulgaris* (sugar beet), charcoal rot

## Abstract

‘*Candidatus* Phytoplasma solani’ (stolbur phytoplasma) is associated with rubbery taproot disease (RTD) of sugar beet (*Beta vulgaris* L.), while *Macrophomina phaseolina* is considered the most important root rot pathogen of this plant in Serbia. The high prevalence of *M*. *phaseolina* root rot reported on sugar beet in Serbia, unmatched elsewhere in the world, coupled with the notorious tendency of RTD-affected sugar beet to rot, has prompted research into the relationship between the two diseases. This study investigates the correlation between the occurrence of sugar beet RTD and the presence of root rot fungal pathogens in a semi-field ‘*Ca*. P. solani’ transmission experiment with the cixiid vector *Reptalus quinquecostatus* (Dufour), in addition to naturally infected sugar beet in the open field. Our results showed that: (i) *Reptalus quinquecostatus* transmitted ‘*Ca*. P. solani’ to sugar beet which induced typical RTD root symptoms; (ii) *Macrophomina phaseolina* root rot was exclusively present in ‘*Ca*. P. solani’-infected sugar beet in both the semi-field experiment and naturally infected sugar beet; and that (iii) even under environmental conditions favorable to the pathogen, *M*. *phaseolina* did not infect sugar beet, unless the plants had been previously infected with phytoplasma.

## Introduction

Rubbery taproot disease (RTD) of sugar beet in Serbia and the Pannonian Plain has been associated with the plant pathogenic microorganism ‘*Candidatus* Phytoplasma solani’ (Mollicutes, Acholeplasmataceae) (Quaglino et al., 2013; Ćurčić et al., 2021a,b). ‘*Candidatus* P. solani,’ known by its trivial name “stolbur phytoplasma,” is a fastidious, phloem-limited bacterium that infects a variety of cultivated plants across Europe, occasionally causing serious economic losses (Mitrović et al., 2013; EPPO, 2023). Several insect species of the family Cixiidae (Hemiptera, Auchenorrhyncha) have been identified as vectors of ‘*Ca*. P. solani’ (Jović et al., 2019; Kosovac et al., 2023). A particular cixiid planthopper, *Reptalus quinquecostatus* (Dufour) *sensu* Holzinger et al. (2003), has recently been revealed as a ‘*Ca*. P. solani’ vector to sugar beet in Serbia and proposed culpable for the 2020 epidemic RTD outbreak recorded in Rimski Šančevi (Novi Sad, northern Serbia) (Kosovac et al., 2023). Symptoms of sugar beet RTD first appear in the second half of July, approximately a month after ‘*Ca*. P. solani’ has been transmitted by vector(s) in the field. The symptoms begin with a loss of turgor in leaves during the hottest part of the day, followed by yellowing and necrosis of the oldest leaves. Eventually, all leaves become necrotic, which leads to the complete decline of the plant. At the same time, taproots of diseased plants wilt and become rubbery. Although initially without any rot symptoms, taproots begin to rot after aboveground parts of the plant have declined. As a consequence, some of the taproots completely rot before harvest (Ćurčić et al., 2021b; Kosovac et al., 2023).

Among other reported pathogens (*Fusarium* spp.*, Rhizoctonia solani*)*, Macrophomina phaseolina* (Tassi) Goid (Botryosphaeriaceae) is currently considered the most important root rot fungal pathogen of sugar beet in Serbia. In extreme environmental conditions (i.e., warm summers and severe droughts), it may cause losses of up to 100% (Jasnić et al., 2005; Stojšin et al., 2012; Budakov et al., 2015). *Macrophomina phaseolina* is a soil-borne, necrotrophic pathogen present all across the world, affecting more than 500 plant species (100 families) (Babu et al., 2007; Abass et al., 2021; Marquez et al., 2021). It is the causal agent of stem and root rot, seedling blight and charcoal rot. *Macrophomina phaseolina* survives for at least 2 years as sclerotia, formed in host plants, soil or leftover host tissue (Collins, 1988; Su et al., 2001). The fungus prefers temperatures in the range of 30–35°C, though some isolates have the greatest growth rate at 40°C (Manici et al., 1995). Under the conditions of high temperatures (30–35°C) and low soil moisture (below 60%), *M*. *phaseolina* may cause significant yield losses in soybean and sorghum. In extreme cases, 100% yield losses have been recorded in groundnut cultivars when the disease appeared at the pre-emergence stage (Kaur et al., 2012; Marquez et al., 2021). Taxonomically, *M*. *phaseolina* had been the only species in the genus *Macrophomina*, until recently when multilocus phylogenetic analysis allowed the description and distinction of four cryptic *Macrophomina* species—*M*. *pseudophaseolina*, *M*. *euphorbiicola*, *M*. *vaccinii*, and *M*. *tecta* (Sarr et al., 2014; Machado et al., 2019; Zhao et al., 2019; Poudel et al., 2021).

In addition to Serbia*, M*. *phaseolina* in sugar beet has been reported in the hot inland valleys of California (United States), India, Iran, Egypt, Russia, and some other countries of the former USSR, Greece, and Hungary. In these countries, it is generally considered a minor root rot pathogen of weakened, injured or stressed plants (Cooke and Scott, 1993; Karadimos et al., 2002; Jacobsen, 2006). Recent studies of microbial communities in both healthy and root rot-affected sugar beet in Austria and Germany, using conventional (isolation) and molecular techniques (including high-throughput sequencing), found *M*. *phaseolina* neither in healthy nor root rot-affected sugar beet, unlike other pathogenic or nonpathogenic fungi (Liebe et al., 2016; Liebe and Varrelmann, 2016; Kusstatscher et al., 2019).

The observed tendency of ‘*Ca*. P. solani’-infected sugar beet to rot, as well as the high prevalence of *M*. *phaseolina* root rot reported in sugar beet in Serbia (compared to its negligible impact in other regions across the world) prompted investigation into the relationship between the presence of ‘*Ca*. P. solani’ and root rot fungi in sugar beet in Serbia. Therefore, the aim of this interdisciplinary study was: (i) to study the correlation between the occurrence of RTD of sugar beet and the presence of root rot fungal pathogens in a semi-field ‘*Ca*. P. solani’ transmission experiment with vector *R*. *quinquecostatus sensu* Holzinger et al. (2003); (ii) to further assess and confirm the dominance of *M*. *phaseolina* root rot in ‘*Ca*. P. solani’-infected sugar beet in open-field conditions; and (iii) to characterize selected isolates of ‘*Ca*. P. solani’ on the epidemiologically informative *tuf* and *stamp* genes, and to morphologically and molecularly characterize *M*. *phaseolina*.

## Materials and methods

### Semi-field ‘*Candidatus* Phytoplasma solani’ transmission experiment

Our study of the relationship between ‘*Ca*. P. solani’ infection of sugar beet and fungal root rot was conducted from May to November 2022, at a long-term experimental field in Rimski Šančevi (N 45°19´57″; E 19°49′58″) at the Institute of Field and Vegetable Crops, Novi Sad. The long-term experimental field was set up in 1965 as a four-field crop rotation scheme for sugar beet, corn, sunflower, and wheat, 2 ha each. For the semi-field experiment, two net cages (2 m × 2 m × 2.5 m) were installed in the sugar beet plot on May 15, covering 40 plants each, and subjected to the same agrotechnical protocol as the rest of the field. The aim of the semi-field experiment was to ensure a pool of RTD-affected sugar beet using a naturally infected population of a certain cixiid vector present *in situ*. An abundant population of *Reptalus* sp. aggregated in Rimski Šančevi on a parsnip field bordering the experimental sugar beet plot. When the first adults appeared at the beginning of June 2022, a total of 30 insects were caught. Species identity of collected males was determined by a specific morphological difference in the anal tube—a distinct process with a left orientation in *R*. *quinquecostatus*, but absent in its congeneric species *R*. *panzeri* (Holzinger et al., 2003). Genomic DNA was isolated from individual insects using a modified CTAB method (Gatineau et al., 2001), primarily to molecularly determine the identity of sampled females based on the internal transcribed spacer 2 (ITS2) (Bertin et al., 2010; Kosovac et al., 2023). After all 30 representative individuals were identified as *R*. *quinquecostatus sensu* Holzinger et al. (2003), insects were subjected to ‘*Ca*. P. solani’ detection to confirm the infection status of the targeted population. Detection was performed by amplifying the ‘*Ca*. P. solani’—specific *stamp* gene in nested PCR assays, using Stamp-F/R0 and Stamp-F1/R1 primer pairs and following previously described conditions (Fabre et al., 2011). Each 25 μL PCR mix contained 20 ng of template DNA, 1× PCR Master Mix (Thermo Scientific, Vilnius, Lithuania) and 0.4 μM of each primer. Samples lacking template DNA were employed as negative controls. In total, 1 μL of direct PCR amplicon diluted 30× in sterile water was used as a template for nested PCR. Six microlitres of nested PCR products were then separated in a 1% agarose gel, stained by ethidium bromide, and visualized with a UV transilluminator. Amplification of the fragment of expected size, ~470 bp, was considered a positive reaction. A total of 250 *R*. *quinquecostatus* individuals, collected shortly afterward from the assessed population, were released on June 9, 2022, into one of the two net cages described above, whereas the other cage without insects was used as a negative control.

Sugar beets in the semi-field experiment were visually evaluated for the development of RTD leaf symptoms once a week or more frequently. Sampling of the sugar beet root tissue was done depending on RTD and rot symptom severity and plant decline. The final sampling was done in the beginning of October 2022. All 80 experimental sugar beet from both cages, RTD and root rot-symptomatic, as well as the asymptomatic plants, were further subjected to phytoplasma and fungi assessment.

### Open-field sugar beet assessment

Sampling of open-field sugar beet was conducted during November 2022 at three locations: Rimski Šančevi, where the semi-field experiment was performed, Banatsko Veliko Selo (N 45°47′56″; E 20°34′43″; ~80 km north-east of the experimental field) and Sremska Mitrovica (N 44°57′20″; E 19°40′24″; ~45 km south-west). A total of 180 sugar beet samples (60 per each field) were collected: (1) 20 with prominent RTD symptom rubbery taproot, but without rot; (2) 20 with charcoal root rot; and (3) 20 asymptomatic (without RTD and root rot). All field-collected samples were further subjected to phytoplasma and fungi assessment as described onward.

### Phytoplasma assessment

Nucleic acid extraction from all sugar beet samples (semi-field and open-field) was performed from 0.5 g of taproot tissue, following the CTAB protocol (Doyle and Doyle, 1990). Total nucleic acids were precipitated with isopropanol, re-suspended in TE buffer (10 mM Tris pH 8 and 1 mM EDTA) and stored at −20°C.

For phytoplasma assessment in collected samples, amplification of the ‘*Ca*. P. solani’—specific *stamp* gene was performed in nested PCR assays as described above. To examine the presence of phytoplasmas other than ‘*Ca*. P. solani,’ samples evaluated as negative in *stamp* PCR, were further subjected to a universal phytoplasma assay using the TaqMan real-time PCR protocol (qPCR), which targets the 16S rRNA gene of phytoplasmas and the 18S rRNA gene of plants (to confirm the presence of the DNA template and evaluate its quality) as described by Christensen et al. (2004, 2013) with a few modifications. Briefly, the final reaction volumes of 15 μL contained 1x TaqMan qPCR master mix (Nippon genetics Europe), 1 μL template DNA, 0.15 μL Uracil-N-Glycosylase (UNG), and 0.4 μM of each primer and probe. The qPCR was performed in a Magnetic Induction Cycler, Mic (Bio Molecular Systems, Upper Coomera, Australia). Each assay included a DNA-free blank reaction, a negative control corresponding to an RTD asymptomatic sugar beet, and a positive control of ‘*Ca*. P. solani,’ strain 284/09 (Mitrović et al., 2014). Data evaluation was performed using micPCR^©^ software Version 2.6.4 (Bio Molecular Systems, Upper Coomera, Australia).

All ‘*Ca*. P. solani’-positive samples were further subjected to characterization of the epidemiologically decisive *tuf* gene that indicates strains affiliation to a specific epidemiological cycle (Langer and Maixner, 2004; Aryan et al., 2014; Ćurčić et al., 2021b). To amplify the *tuf* gene, the Tuf1-f1/Tuf1-r1 (CACGTTGATCACGGCAAAAC/CCACCTTCACGGATAGAAAAC) and fTufAy/rTufAy primer pairs were used in nested PCR assays (Schneider and Gibb, 1997; Langer and Maixner, 2004; Kosovac, 2018). For differentiation of the *tuf* types (tuf-a, b, and d), the obtained *tuf* amplicons (fTufAy/rTufAy) were subjected to RFLP analyses with *Hpa*II and *Tai*I restriction enzymes (Thermo Scientific) in separate reactions, according to manufacturer’s instructions (Langer and Maixner, 2004; Ćurčić et al., 2021b). Restriction products were separated in an 8% polyacrylamide gel, stained and visualized as described above. To check for the presence of additional variability in the *tuf* gene, six randomly selected sugar beets from the semi-field transmission experiment and from each of the assessed open fields (24 in total) were subjected to *tuf* gene sequence analyses. The fTufAy/rTufAy nested PCR products were sequenced in both directions with the primers applied for amplification, to yield a 2X consensus amplicon sequence, using a commercial service (Macrogen Inc., Seoul, Korea). The *tuf* sequences were then assembled using Pregap4 from the Staden program package (Staden et al., 2000) and subjected to multiple sequence alignment using ClustalX in MEGA X (Thompson et al., 1997; Kumar et al., 2018). Strains CrHo13_1183, CrHo12_601, CrHo12_650, and 429/19 corresponding to the previously described ‘*Ca*. P. solani’ *tuf* genotypes tuf a, tuf b1, tuf b2, and tuf d, respectively (Aryan et al., 2014; Ćurčić et al., 2021b), were used for the comparison.

In all 24 ‘*Ca*. P. solani’ strains selected for *tuf* gene sequence analyses, *stamp* gene was also sequenced in both directions as described above since its diversity follows up epidemiological divergence that *tuf* gene basically reveals (Fabre et al., 2011). The obtained sequences were assembled using Pregap4 from the Staden program package (Staden et al., 2000), manually inspected and compared with those of the publicly available strains representing previously described *stamp* genotypes (Pierro et al., 2018) using BLAST in the GenBank.

### Fungal assessment

Sugar beet roots with two types of symptoms: root rot and rubbery taproots without rot, as well as asymptomatic roots, were assessed for the presence of fungi. Isolation was done from the margin of healthy and rotted tissue of roots with rot symptoms and from the internal portion of the roots without rot (rubbery taproot and asymptomatic). Root fragments were washed, disinfected in 70% ethanol and placed on potato dextrose agar (PDA, EMD, Darmstadt, Germany, pH 5.6 ± 0.2) in Petri dishes (90 mm). After 3–5 days of incubation at 24 ± 2°C in 12/12 h light/dark regime, developing fungal colonies were transferred to a pure culture and their morphology was assessed. Isolates with colony features typical for *Macrophomina* sp. (initially whitish colonies that become dark grey with age and develop numerous black sclerotia) (Sarr et al., 2014) were further subjected to molecular analyses for fungal species confirmation, whereas other isolates were identified at genus level based on morphology.

DNA was extracted from 7-day-old cultures of obtained isolates, according to the previously described CTAB protocol (Day and Shattock, 1997). The isolates were tested using *M*. *phaseolina*—specific primers for translation elongation factor 1α (TEF1-α) MpTefF/MpTefR, following previously described conditions (Santos et al., 2020). Amplification of the fragment of expected size, ~220 bp, was considered a positive reaction. A total of seven *M*. *phaseolina* isolates, two per open-field locality and one from the semi-field experiment, were randomly selected for further molecular and morphological characterization. Five loci selected for characterization—internal transcribed spacer regions 1 and 2, including the 5.8S rRNA gene (ITS), translation elongation factor 1-α (TEF1-α), actin (ACT), calmodulin (CAL), and β-tubulin (TUB) genes—were amplified using primer pairs ITS1/ITS4 (White et al., 1990), EF1-728F (Carbone and Kohn, 1999)/EF2R (Jacobs et al., 2004), ACT-512F/ACT-783R (Carbone and Kohn, 1999), CAL-228F/CAL-737R (Carbone and Kohn, 1999), and T1 (O’Donnell and Cigelnik, 1997)/Bt2b (Glass and Donaldson, 1995), respectively. The PCR conditions were as follows: initial denaturation at 95°C for 2 min, followed by 35 cycles of denaturation at 95°C for 30 s, annealing at 52°C for 30 s (ITS), or 55°C for 50 s (CAL), or 55°C for 1 min (TEF1-α, ACT, and TUB), and elongation at 72°C for 1 min, and a final elongation at 72°C for 10 min. Each 25 μL PCR mix contained 20 ng of template DNA, 1× PCR Master Mix (Thermo Scientific, Vilnius, Lithuania), and 0.4 μM of each primer. Samples lacking template DNA were employed as negative controls. PCR products (5 μL) were separated in a 1.5% agarose gel, stained and visualized as described above. Amplified products were purified and sequenced in both directions as described above. Sequences were assembled and deposited in the NCBI GenBank. Evolutionary history was inferred based on combined analyses of the five loci (ITS, TEF-1α, ACT, CAL, and TUB) of seven isolates obtained in this study, reference isolates of *Macrophomina* spp. and *Botryosphaeria dothidea* CBS115476 as an outgroup (Supplementary Table S1), using the Maximum Likelihood (ML) and Maximum Parsimony (MP) methods (MEGA X). For ML, the best nucleotide substitution model was determined using the “find best model” option in MEGA X. Initial tree(s) for the heuristic search were obtained automatically by applying Neighbor-Join and BioNJ algorithms to a matrix of pairwise distances estimated using the Maximum Composite Likelihood approach, and then selecting the topology with superior log likelihood value. The MP trees were obtained using the Tree-Bisection-Reconnection (TBR) algorithm with search level 3, in which the initial trees were obtained by the random addition of sequences (10 replicates). To estimate the statistical significance of the inferred clades, 1,000 bootstraps were performed.

Morphological characterization of the seven selected isolates was performed on PDA at 24°C for 3 days in the dark for macromorphology and on pine needle agar (PNA) at 24°C under 12/12 h light/dark regime for 4–8 weeks for micromorphology (Crous et al., 2006; Sarr et al., 2014). Morphology of sclerotia, conidiomata, and conidia was evaluated using the compound microscope Zeiss Axio Lab, Jena, Germany. Photographs and measurements were obtained using the camera Axiocam ERc 5 s, Zeiss and software ZEN 2 (blue edition), Jena, Germany.

## Results

### *Reptalus quinquecostatus* transmits ‘*Candidatus* Phytoplasma solani’ to sugar beet, RTD develops, and root rot follows

Combination of morphology and molecular tools applied in identification of the 30 *Reptalus* sp. individuals (19 males and 11 females), sampled prior to the set-up of the semi-field experiment, confirmed presence of only *R*. *quinquecostatus sensu* Holzinger et al. (2003) aggregating on the bordering parsnip. As the ‘*Ca*. P. solani’ infection rate of the analyzed *R*. *quinquecostatus* population was 63% which indicated its high potential to experimentally induce RTD in sugar beet, this population was further used in the semi-field sugar beet experiment.

**Figure 1:**
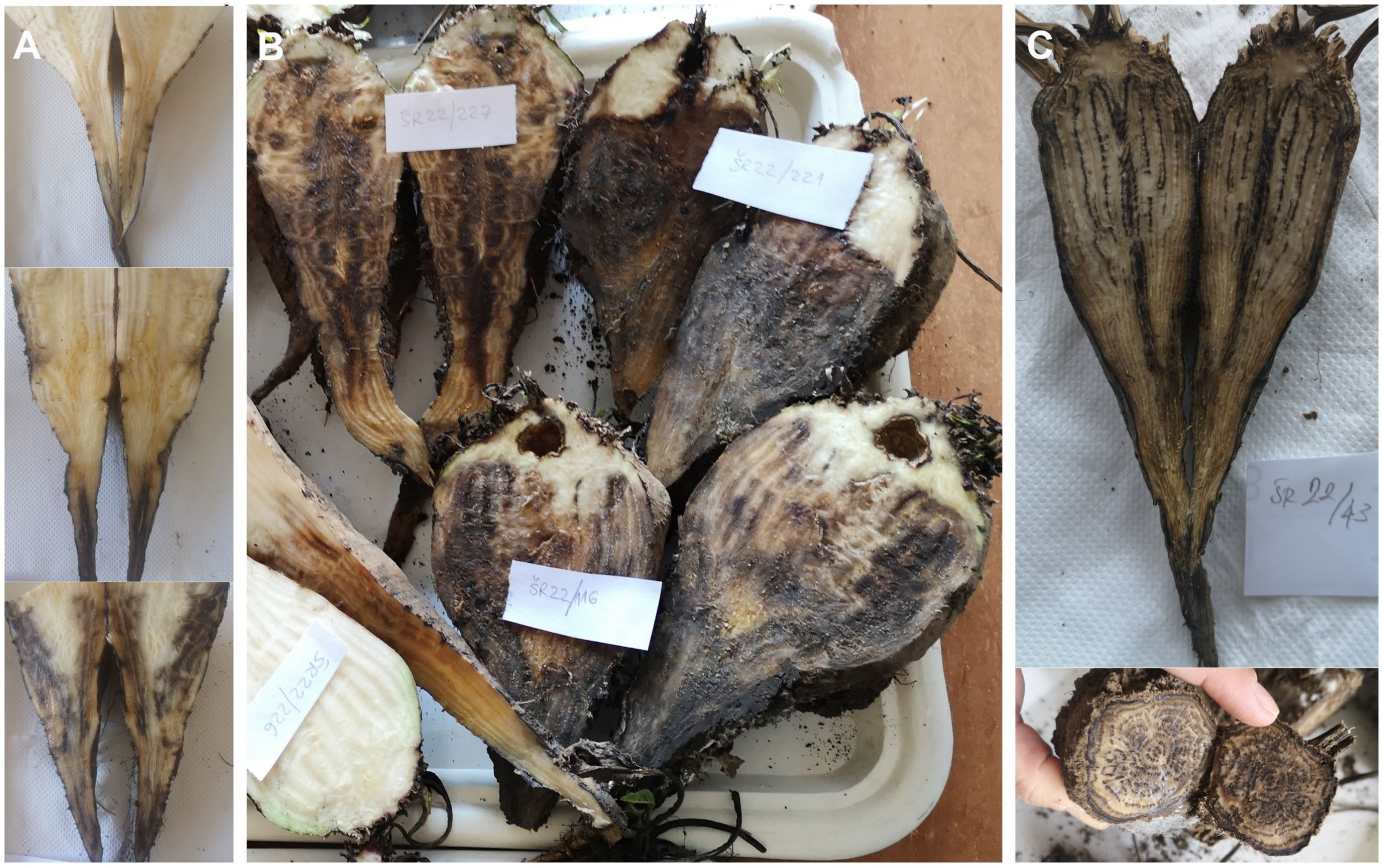
Cross section of sugar beet infected with ‘*Candidatus* Phytoplasma solani’ and *Macrophomina phaseolina*. **(A)** Early stage of charcoal root rot beginning from root tail; **(B)** Different stages of charcoal root rot; and **(C)** Advanced stage of charcoal root rot.

The first RTD symptomatic sugar beet in the cage with released *R*. *quinquecostatus* were observed in mid-July (45 DAI). The symptoms included loss of turgor in leaves during the hottest part of the day, followed by yellowing and, later, necrosis of the oldest leaves, progressing from their margins. Eventually, all leaves became necrotic, which led to the decline of the plants. Out of 40 sugar beet exposed to *R*. *quinquecostatus*, 32 declined plants were collected on August 10 (62 DAI). The remaining eight plants (of which one had declined, three presented RTD leaf symptoms and four were asymptomatic), were finally collected on September 8 (91 DAI). The declined sugar beets exhibited different stages of charcoal root rot with root tissue color varying from light yellow and brown to black on cross section, usually starting from the tail (Figure 1). Some of the declined sugar beets had advanced stage of root rot and hence it was challenging to evaluate rubberiness of their taproots, whereas some of the declined plants with rubbery taproots had early stage of root rot, clearly visible just after cutting the taproot (Figure 1). The three sugar beets with RTD leaf symptoms had rubbery taproots without root rot, which on cross section were visually indistinguishable from healthy taproots and lacked discoloration. The four sugar beets collected as asymptomatic had neither rubbery taproots nor root rot. In the control cage without insects, all 40 sugar beets remained RTD-asymptomatic on their leaves and were collected in the beginning of October as free of rubbery taproots and root rot. Molecular analysis of sugar beet samples from the cage with *R*. *quinquecostatus* revealed ‘*Ca*. P. solani’ infection in 36 out of 40 sugar beets, including all 33 declined plants and three RTD symptomatic lacking root rot. The remaining four asymptomatic sugar beet, as well as all 40 asymptomatic sugar beets (no RTD or root rot) from the control cage resulted negative in both the ‘*Ca*. P. solani’—specific PCR and universal phytoplasma qPCR assays (Table 1).

**Table 1:** Sugar beet root symptoms and presence of ‘*Ca*. P. solani’ and *M*. *phaseolina* in the semi-field experiment with *R*. *quinquecostatus*.

Semi-field trial	*R*. *quinquecostatus* test cage			Negative control cage
Symptoms	RTD + root rot 33/40^*^	RTD 3/40	Asymp 4/40	Asymp 40/40
‘*Ca*. P. solani’	33/33	3/3	0/4	0/40
*M*. *phaseolina*	33/33	0/3	0/4	0/40
Other fungi^**^	0/33	0/3	1/4 Fus	8/40 Fus6/40 Rhi4/40 Pen

* Number of samples in which the symptom or pathogen is present/total number of assessed.

** Fus, *Fusarium* sp.; Pen, *Penicillium* sp.; Rhi: *Rhizopus* sp.

### *Macrophomina phaseolina* is present only in root rot of sugar beet with ‘*Candidatus* Phytoplasma solani’

Among sugar beet from the *R*. *quinquecostatus* transmission cage, fungal assessment revealed the presence of *M*. *phaseolina* in all 33 declined plants, which were also ‘*Ca*. P. solani’-infected, whereas no *M*. *phaseolina* presence was confirmed in the seven sugar beet without rot, regardless of phytoplasma presence. Neither was *M*. *phaseolina* presence confirmed in any of the 40 asymptomatic sugar beet from the negative control cage. In sugar beet without ‘*Ca*. P. solani’ infection, fungi other than *M*. *phaseolina* were sporadically isolated (*Fusarium* sp., *Penicillium* sp. and *Rhizopus* sp.; Table 1).

Similar to the semi-field experiment, samples collected from the open fields with charcoal root rot expressed also rubberiness, although evaluating rubberiness of the taproots was challenging in the declined sugar beet with advanced stage of root rot. Presence of ‘*Ca*. P. solani’ followed the same occurrence pattern in the open-field samples as in the semi-field transmission experiment at each of the three assessed localities: 60 declined sugar beet with charcoal root rot and 60 RTD symptomatic ones (with rubbery, but not rotted taproots) were positive for ‘*Ca*. P. solani,’ whereas all 60 asymptomatic sugar beets were negative for ‘*Ca*. P. solani’ and universal phytoplasma (Table 2). Similarly, results of fungal assessment of open-field samples were comparable with results obtained in the semi-field experiment. *Macrophomina phaseolina* was detected in all 60 declined sugar beets with charcoal root rot, but not in any of the 60 rubbery taproot sugar beets without root rot or in any of the 60 asymptomatic plants regardless of phytoplasma presence. Moreover, as in the semi-field transmission experiment, in rubbery taproot sugar beet without rot and asymptomatic sugar beet, fungi other than *M*. *phaseolina* from the same genera (*Fusarium* sp., *Penicillium* sp. and *Rhizopus* sp.) were sporadically isolated regardless of phytoplasma presence (Table 2).

**Table 2:** Sugar beet root symptoms and presence of ‘*Ca*. P. solani’ and *M*. *phaseolina* in the open field.

Locality	Rimski Šančevi			Banatsko Veliko Selo			Sremska Mitrovica		
Symptoms	RTD + root rot	RTD	Asymp	RTD + root rot	RTD	Asymp	RTD + root rot	RTD	Asymp
‘*Ca*. P. solani’	20/20^*^	20/20	0/20	20/20	20/20	0/20	20/20	20/20	0/20
*M*. *phaseolina*	20/20	0/20	0/20	20/20	0/20	0/20	20/20	0/20	0/20
Other fungi^**^	0/20	9/20 Fus1/20 Rhi1/20 Pen	6/20 Fus2/20 Rhi	0/20	5/20 Fus2/20 Rhi	8/20 Fus	0/20	5/20 Fus4/20 Rhi7/20 Pen	3/20 Fus3/20 Rhi4/20 Pen

* Number of samples in which the pathogen is present/total number of assessed.

** Fus: *Fusarium* sp.; Pen: *Penicillium* sp.; Rhi: *Rhizopus* sp.

### Molecular characterization of ‘*Candidatus* Phytoplasma solani’

**Figure 2:**
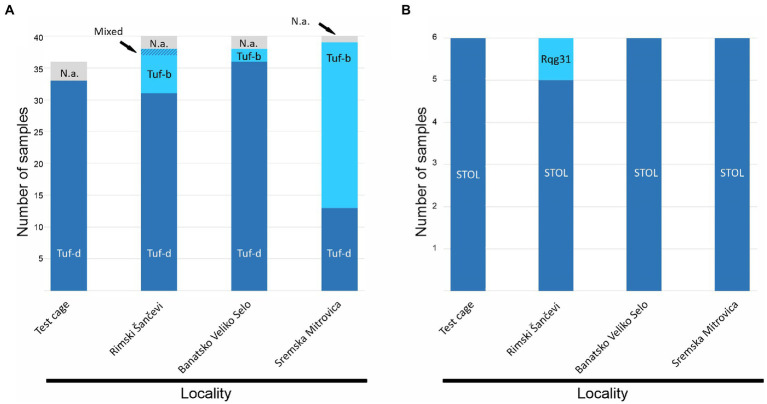
Molecular characterization of ‘*Candidatus* Phytoplasma solani’ based on **(A)** *tuf* gene **(B)** *stamp* gene. N.a. not amplified.

The expected *tuf* gene amplicons were obtained for 148 out of 156 ‘*Ca*. P. solani’ infected sugar beets (36 RTD symptomatic plants from the semi-field experiment and 120 plants from the open-field assessment). *Tuf* gene RFLP analyses revealed the presence of the tuf-d type in 33 out of 36 RTD symptomatic and ‘*Ca*. P. solani’ positive sugar beets from the *R*. *quinquecostatus* transmission experiment that were amplified on the *tuf* gene, while in samples collected in field, the tuf-b type was also recorded. In Rimski Šančevi, 31 out of 38 sugar beet samples assessed for the *tuf* gene had the tuf-d type, six had the tuf-b type, while one sample showed mixed infection with the two tuf types. In Banatsko Veliko Selo the tuf-d type also dominated in analyzed samples and was found in 36 out of 38 sugar beet samples, with the tuf-b type present in only two sugar beets. In Sremska Mitrovica, the majority of the analyzed plants, 26 out of 39, had tuf-b, whereas the tuf-d type was present in 13 sugar beet. Sequencing of the 24 randomly selected strains confirmed the presence of the tuf-d genotype in 23 out of 24 analyzed samples, whereas the tuf-b1 genotype was found in one sample from the field in Rimski Šančevi (Figure 2A).

The partial *stamp* gene sequences obtained from the same set of samples showed prevalence of the STOL (St4) *stamp* genotype in 23 out of 24 samples, whereas in Rimski Šančevi, only one sugar beet, with the tuf-b type, had the Rqg31 (St2) genotype (Figure 2B).

### Molecular and morphological characterization of *Macrophomina phaseolina*

**Figure 3:**
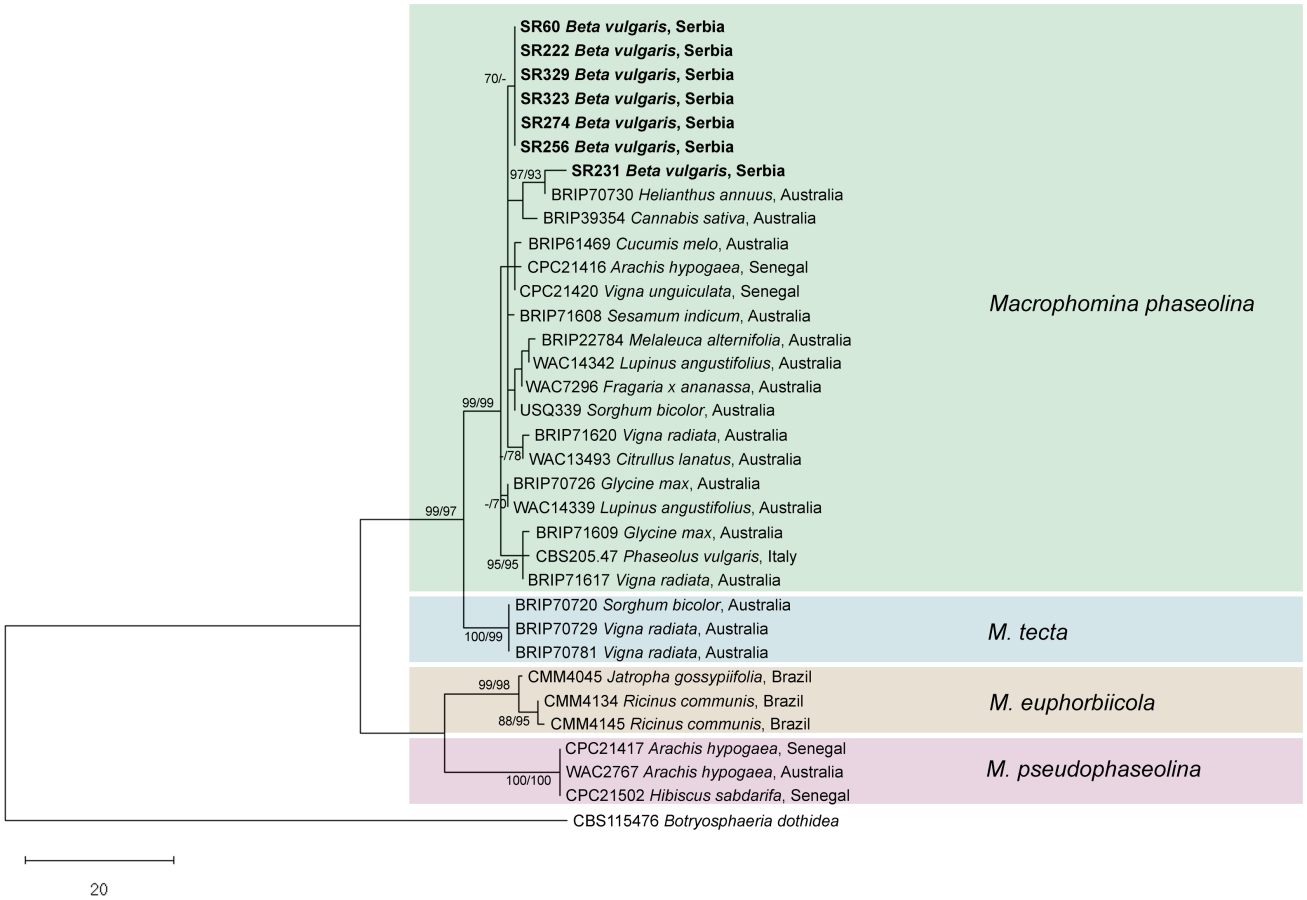
Phylogenetic tree resulting from the analysis of concatenated ITS, TEF1-α, ACT, CAL, and TUB sequences of *Macrophomina* spp. Numbers on the branches represent maximum parsimony and maximum likelihood bootstrap values (MP/ML) from 1,000 replicates. Values less than 70% are marked with “-.” The tree was rooted to *Botryosphaeria dothidea*. The scale bar represents 20 nucleotide substitutions. Isolates obtained in this work are shown in bold.

In all fungal isolates forming dark grey colonies with numerous black sclerotia on PDA, *M*. *phaseolina* was confirmed with *M*. *phaseolina*-specific primers (MpTefF/MpTefR) that generated amplicons of ⁓220 bp in PCR, whereas no amplification was observed in the negative controls. ITS, TEF1-α, ACT, CAL, and TUB amplicons of expected size (⁓600, 300, 300, 580, and 700 bp, respectively) were obtained for the seven selected isolates. Sequencing of the obtained amplicons yielded nucleotide sequences of 544–545 nt for ITS, 259–260 nt for TEF1-α, 260 nt for ACT, 544 nt for CAL, and 650 nt for TUB, which were deposited in the NCBI GenBank^1^ under accession numbers provided in Supplementary Table S1. Six out of seven analyzed isolates were identical in all five assessed loci, while one (SR231) differed from the other six in all loci (2 nt in ITS and TEF1-α, 1 nt in ACT, 3 nt in CAL, and 4 nt in TUB). The combined dataset of the concatenated five locus alignments contained 2,064 characters, of which 79 were parsimony informative. MP analysis resulted in eight equally most parsimonious trees. The phylogenetic tree constructed by the ML method, using the Hasegawa-Kishino-Yano model, had the same topology as the MP tree. A representative phylogenetic tree is presented in Figure 3. Multilocus phylogeny confirmed the identity of the obtained isolates as *M*. *phaseolina* (Figure 3). Six isolates from sugar beet formed a subclade within *M*. *phaseolina*, while one isolate (SR231) clustered separately with the *M*. *phaseolina* isolate from *Helianthus annuus* from Australia (BRIP70730), from which it differed in 3 nt in CAL.

**Figure 4:**
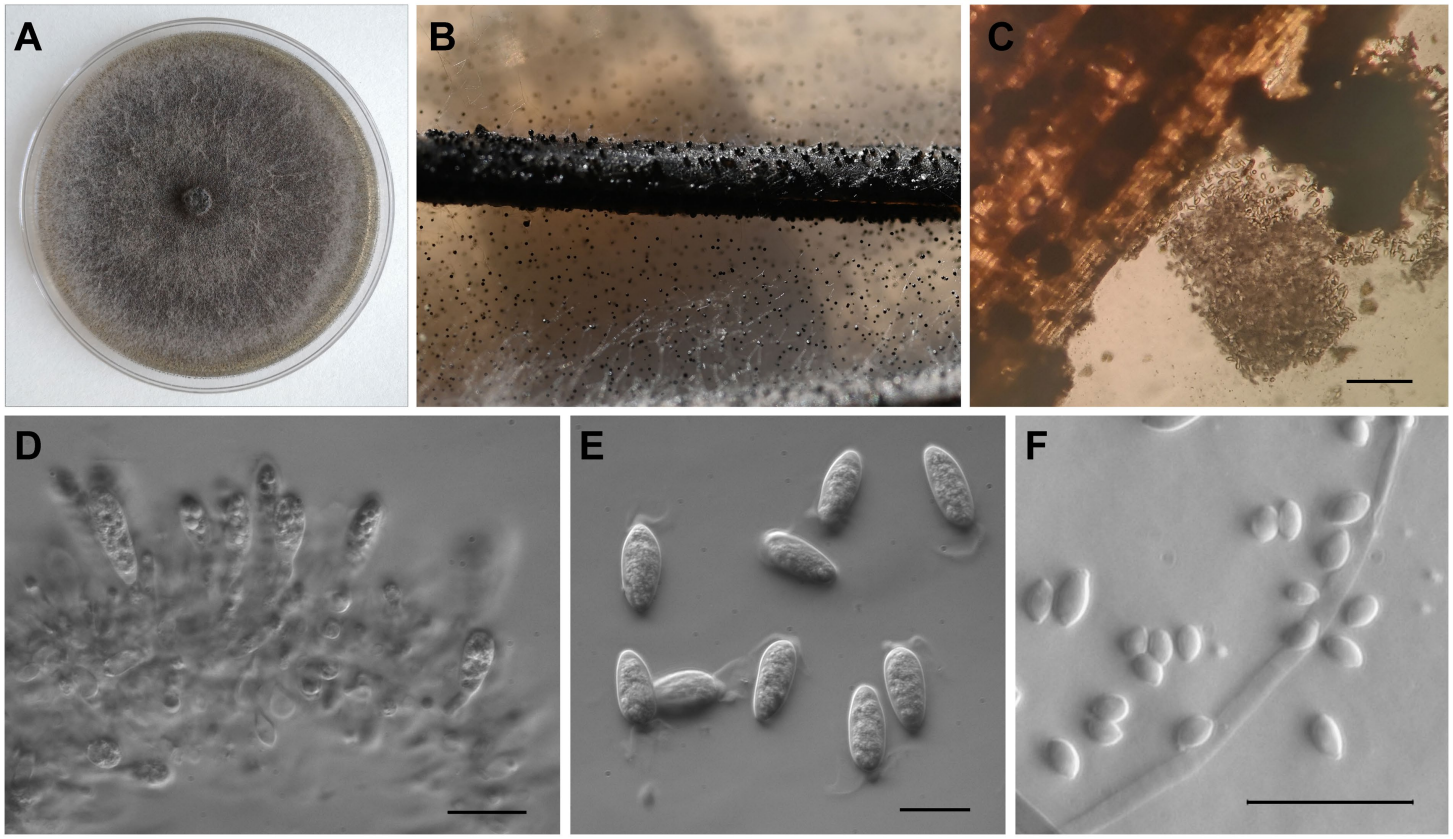
Morphological characteristics of *Macrophomina phaseolina* isolated from sugar beet in Serbia. **(A)** Colony on PDA; **(B)** Sclerotia and conidiomata on PNA; **(C)** Conidiomata and conidia; **(D)** Conidiogenous cells; **(E)** Conidia with apical appendages; and **(F)** Microconidia. In **(C)** scale bar = 200 μm, while in **(D–F)** scale bar = 20 μm.

*Macrophomina phaseolina* colonies had even margins, were initially white with an abundant fluffy or flat aerial mycelium, and turned dark grey with age, developing dense, black sclerotial masses on PDA (Figure 4A). After 3 days on PDA, the average colony diameter was 68.11 ± 1.84 mm. Sclerotia were black, smooth, and hard (mean diam. ± SE of 169 sclerotia 108.5 ± 2.1 μm; Figure 4B). Conidiomata were dark brown to black, solitary or gregarious (Figures 4B,C). Conidiogenous cells were hyaline, short, obpyriform to subcylindrical (Figure 4D). Conidia (Figure 4E) were ellipsoid to obovoid, hyaline and with apical mucoid appendages, (20.82–) 23.78–26.48 (−30.19) μm long and (8.8–) 9.95–10.88 (−11.72) μm wide (mean ± SE of 100 conidia = 25.15 ± 0.2 × 10.4 ± 0.06 μm). Microconidia were aseptate, hyaline and smooth (mean ± SE of 30 microconidia = 5.8 ± 0.13 × 3.5 ± 0.12 μm; Figure 4F).

## Discussion

This study investigates the relationship between the presence of fungal root rot pathogens and occurrence of ‘*Ca*. P. solani’-associated RTD of sugar beet in Serbia. In both the semi-field experiment and open-field assessment, *M*. *phaseolina* was found only in ‘*Ca*. P. solani’-infected sugar beet. Apart from *M*. *phaseolina*, which was predominant on RTD-affected sugar beet with root rot, few other fungi were found in sugar beet without root rot regardless of phytoplasma presence or RTD symptoms. *Macrophomina phaseolina* has been reported as the most significant root rot pathogen of sugar beet in Serbia, causing economic damage that exceeds the impact of other fungal pathogens (Budakov et al., 2015). On the other hand, ‘*Ca*. P. solani’ causes the typical RTD symptom, rubbery taproot, which facilitates rotting of sugar beet, as reported in current and previous studies (Ćurčić et al., 2021a,b). Accordingly, a common trait of both sugar beet pathogens—to escalate during warm droughty summers (Marić, 1974; Budakov et al., 2015; Ćurčić et al., 2021b)—suggests a plausible correlation that has not been investigated to date.

Results obtained in the semi-field transmission experiment in Rimski Šančevi, involving *R*. *quinquecostatus sensu* Holzinger et al. (2003), corroborated the ‘*Ca*. P. solani’ vectoring role of this cixiid planthopper in the sugar beet RTD context (Kosovac et al., 2023). The transmission experiment with *R*. *quinquecostatus* resulted in 90% ‘*Ca*. P. solani’ infection rate of sugar beet in the experimental cage. Typical leaf RTD symptoms such as loss of turgor, wilting, yellowing, and necrosis, were previously reproduced in laboratory-controlled single-plant experiments using this insect vector, but ruberiness of the taproot had not developed in the test plants, likely because of an optimal watering regime (Kosovac et al., 2023). However, in the semi-field experiment, 36 out of 40 sugar beet expressed prominent rubbery taproot with or without root rot. Characterization of ‘*Ca*. P. solani’ strains transmitted by *R*. *quinquecostatus* revealed the presence of only the tuf-d type in infected sugar beet, aligning with experimental results from the 2020 epidemic RTD occurrence on the same locality. Furthermore, all six selected strains characterized on the *stamp* gene belonged to the STOL (St4) genotype, previously reported as the only genotype associated with tuf-d (Ćurčić et al., 2021a,b; Kosovac et al., 2023). However, the presence of two tuf-types in the open–field assessment suggests involvement of vector(s) other than *R*. *quinquecostatus*.

Symptoms observed in the ‘*Ca*. P. solani’ transmission cage—development of rubbery taproots, which are initially without rot, but eventually decline and rot—resemble those in the open fields. Root rot of ‘*Ca*. P. solani’ infected sugar beet in the semi-field experiment was solely due to *M*. *phaseolina*. The strict correlation of *M*. *phaseolina* presence with ‘*Ca*. P. solani’ infection, found on three localities in the open-field assessment, shows that *M*. *phaseolina* did not infect phytoplasma-free sugar beet, even under favorable environmental conditions. Our results suggest that *M*. *phaseolina* amplifies sugar beet yield losses initiated specifically by ‘*Ca*. P. solani,’ which can be the reason for the discrepancy between reports of *M*. *phaseolina* as the most significant fungal root pathogen of sugar beet in Serbia and other reports, in which the fungus is described as a minor threat elsewhere (Cooke and Scott, 1993; Jacobsen, 2006; Budakov et al., 2015). RTD-affected sugar beet without root rot can still be used for processing in industry, providing the condition appears in no more than 2% of sugar beet, while root rot is tolerated in no more than 0.5% (National standard SRPS E.B1. 2002; Sugar beet-quality requirements and sampling).

Though our results suggest that ‘*Ca*. P. solani’ infection renders sugar beet more susceptible to *M*. *phaseolina,* the mechanisms of interactions among the two plant pathogens (a biotroph and a necrotroph) and the plant host are currently unknown. However, it is clear that, because of synergistic interactions, the simple sum of single pathogen infections does not produce equally severe disease symptoms as does co-infection. A similar (bacterium-fungus) synergistic interaction, which leads to a disease complex, has been reported in sugar beet for *Leuconostoc* spp. and *R*. *solani* root rot (Strausbaugh, 2016). Moreover, such cases of complex diseases are not uncommon, as numerous disease complexes have been described in other hosts (reviewed in Agrios, 2005; Lamichhane and Venturi, 2015). Whereas RTD is associated exclusively with ‘*Ca*. P. solani,’ charcoal root rot of sugar beet seems to be a complex disease that occurs as a consequence of RTD and is associated with two species belonging to separate phyla—‘*Ca*. P. solani’ and *M*. *phaseolina*. This is the first description of a phytoplasma-fungus disease complex that may have important implications in the development of an effective plant disease management strategy.

Fungi found in asymptomatic sugar beet were comparable to those isolated from sugar beet with RTD (rubbery taproots), but without root rot. This finding confirms the previously established association of RTD solely with ‘*Ca*. P. solani,’ without the involvement of fungi (Ćurčić et al., 2021a,b; Kosovac et al., 2023). Moreover, all of the fungi isolated from the healthy and rubbery sugar beet taproots without root rot in this study (i.e., *Fusarium* sp., *Penicillium* sp., and *Rhizopus* sp.) have already been reported as present in healthy sugar beet, and as postharvest pathogens (Liebe et al., 2016; Liebe and Varrelmann, 2016; Strausbaugh, 2018; Kusstatscher et al., 2019).

Multilocus phylogeny performed in this study resolved the previously described *Macrophomina* species and confirmed identification of sugar beet isolates from Serbia as *M*. *phaseolina*. Two haplotypes of *M*. *phaseolina* were detected in sugar beet from Serbia, which is in agreement with the previously described high level of intraspecific diversity within *M*. *phaseolina* (Poudel et al., 2021). Furthermore, to our knowledge, our research is the first to provide characterization of five loci (ITS, TEF1-α, ACT, CAL, and TUB) of European *M*. *phaseolina*, beside ex-type CBS 205.47 from Italy.

Considering the longevity of *M*. *phaseolina* sclerotia and an almost 60-year-long four-crop (sugar beet, sunflower, corn, and wheat) agricultural system in Rimski Šančevi, with all listed crops having been reported as hosts of this pathogen, it is likely that the experimental field is highly contaminated with the sclerotia of *M*. *phaseolina* (Jacobsen, 2006; Babu et al., 2007; Abass et al., 2021; Marquez et al., 2021). The crop rotation practice applied in the experimental field is similarly applied in the wider area of Serbia, producing an environment that contributes to the problem. The presence of ‘*Ca*. P. solani’ (reservoir host plant(s) and efficient vector(s)), *M*. *phaseolina* contaminated soil and favorable weather conditions (temperature above 30°C and drought) represents a triangle that creates a “perfect storm” of critical factors causing high yield losses in Serbia. The lack of simultaneous impact of all these factors may explain why ‘*Ca*. P. solani’ infection of sugar beet recorded in some other parts of Europe, such as France, Germany, and Austria (Sémétey et al., 2007; Ćurčić et al., 2021a), is not as devastating as in Serbia. However, the situation may differ in the future because of climate change or interference of other secondary pathogen(s).

## Data availability statement

The datasets presented in this study can be found in online repositories. The names of the repository/repositories and accession number(s) can be found at: https://www.ncbi.nlm.nih.gov/genbank/, OQ420603, OQ420617, OQ421259, OQ420624, OQ420610, OQ420604, OQ420618, OQ421260, OQ420625, OQ420611, OQ420609, OQ420623, OQ421265, OQ420630, OQ420616, OQ420608, OQ420622, OQ421264, OQ420629, OQ420615, OQ420607, OQ420621, OQ421263, OQ420628, OQ420614, OQ420606, OQ420620, OQ421262, OQ420627, OQ420613, OQ420605, OQ420619, OQ421261, OQ420626, and OQ420612.

## Author contributions

BD and ND managed the project and drafted the manuscript. ŽĆ and ER set up and maintained the experimental field. AK designed the transmission experiments and identified insects. AK, ŽĆ, ER, and BD conducted transmission experiments. ŽĆ, ER, BD, ND, and IV collected samples. JS, BD, and AK conducted the phytoplasma analyses. IV, NV, and ND conducted the fungi analyses. BD, JS, AK, ND, and IV contributed to the interpretation of the data. BD, ND, IV, and AK wrote the manuscript. All authors contributed to the article and approved the submitted version.

## Funding

This work was supported by Science Fund of the Republic of Serbia, Program IDEAS (grant no. 7753882, Rubbery Taproot Disease of Sugar Beet: Etiology, Epidemiology, and Control-SUGARBETY) and Ministry of Science, Technological Development and Innovation Republic of Serbia (nos. 451-03-47/2023-01/200116, 451-03-47/2023-01/200214, and 451-03-47/2023-01/200032).

## Conflict of interest

The authors declare that the research was conducted in the absence of any commercial or financial relationships that could be construed as a potential conflict of interest.

## Publisher’s note

All claims expressed in this article are solely those of the authors and do not necessarily represent those of their affiliated organizations, or those of the publisher, the editors and the reviewers. Any product that may be evaluated in this article, or claim that may be made by its manufacturer, is not guaranteed or endorsed by the publisher.

## Footnotes

^1^ www.ncbi.nlm.nih.gov/nucleotide
