# Supplementary material for: A biotroph sets the stage for a necrotroph to play: ‘Candidatus Phytoplasma solani’ infection of sugar beet facilitated Macrophomina phaseolina root rot
Source: Front Microbiol. 2023 Apr 20;14:1164035. doi: 10.3389/fmicb.2023.1164035 (PMC10158981; doi:10.3389/fmicb.2023.1164035)
Supplement: Supplementary file 1 [file Table_1.DOCX]

Supplementary Table S1. GenBank accession numbers of the sequences used in the phylogenetic analysis.

| Species | Isolate^a^ | Host | Location | GenBank Accession Numbers | | | | |
| --- | --- | --- | --- | --- | --- | --- | --- | --- |
|  |  |  |  | ACT | CAL | ITS | TEF1-α | TUB |
| *Macrophomina euphorbiicola* | CMM 4045 | *Jatropha gossypifolia* | Brazil | MF457654 | MF457660 | KU058928 | KU058898 | MF457657 |
|  | **CMM 4134** | *Ricinus communis* | Brazil | MF457655 | MF457661 | KU058936 | KU058906 | MF457658 |
|  | CMM 4145 | *R. communis* | Brazil | MF457656 | MF457662 | KU058937 | KU058907 | MF457659 |
| *M. phaseolina* | SR60 | *Beta vulgaris* | Serbia | OQ420603 | OQ420617 | OQ421259 | OQ420624 | OQ420610 |
|  | SR222 | *B. vulgaris* | Serbia | OQ420604 | OQ420618 | OQ421260 | OQ420625 | OQ420611 |
|  | SR256 | *B. vulgaris* | Serbia | OQ420609 | OQ420623 | OQ421265 | OQ420630 | OQ420616 |
|  | SR274 | *B. vulgaris* | Serbia | OQ420608 | OQ420622 | OQ421264 | OQ420629 | OQ420615 |
|  | SR323 | *B. vulgaris* | Serbia | OQ420607 | OQ420621 | OQ421263 | OQ420628 | OQ420614 |
|  | SR329 | *B. vulgaris* | Serbia | OQ420606 | OQ420620 | OQ421262 | OQ420627 | OQ420613 |
|  | BRIP 22784 | *Melaleuca alternifolia* | Australia | MW592039 | MW592119 | MW591611 | MW592200 | MW592280 |
|  | BRIP 39354 | *Cannabis sativa* | Australia | MW592044 | MW592124 | MW591616 | MW592205 | MW592285 |
|  | BRIP 61469 | *Cucumis melo* | Australia | MW592049 | MW592129 | MW591621 | MW592210 | MW592290 |
|  | BRIP 70726 | *Glycine max* | Australia | MW592057 | MW592137 | MW591628 | MW592217 | MW592299 |
|  | BRIP 70730 | *Helianthus annuus* | Australia | MW592109 | MW592191 | MW591630 | MW592268 | MW592355 |
|  | BRIP 71608 | *Sesamum indicum* | Australia | MW592114 | MW592195 | MW591636 | MW592275 | MW592357 |
|  | BRIP 71609 | *Glycine max* | Australia | MW592062 | MW592143 | MW591637 | MW592221 | MW592305 |
|  | BRIP 71617 | *Vigna radiata* | Australia | MW592070 | MW592151 | MW591645 | MW592229 | MW592313 |
|  | BRIP 71620 | *V. radiata* | Australia | MW592073 | MW592154 | MW591648 | MW592232 | MW592316 |
|  | **CBS 205.47** | *Phaseolus vulgaris* | Italy | KF951804 | MW592161 | KF951622 | KF951997 | MW592323 |
|  | CPC 21420 | *Vigna unguiculata* | Senegal | KF951857 | KF951940 | KF951717 | KF952088 | KF952178 |
|  | CPC 21416 | *Arachis hypogaea* | Senegal | KF951855 | KF951938 | KF951715 | KF952086 | KF952176 |
|  | USQ 339 | *Sorghum bicolor* | Australia | MW592080 | MW592162 | MW591655 | MW592239 | MW592324 |
|  | WAC 13493 | *Citrullus lanatus* | Australia | MW592089 | MW592170 | MW591663 | MW592247 | MW592333 |
|  | WAC 14339 | *Lupinus angustifolius* | Australia | MW592098 | MW592179 | MW591672 | MW592256 | MW592342 |
|  | WAC 14342 | *L. angustifolius* | Australia | MW592101 | MW592182 | MW591675 | MW592259 | MW592345 |
|  | WAC 7296 | *Fragaria x ananassa* | Australia | MW592086 | MW592167 | MW591660 | MW592244 | MW592330 |
| *M. pseudophaseolina* | CPC 21417 | *Arachis hypogaea* | Senegal | KF951918 | KF951986 | KF951791 | KF952153 | KF952233 |
|  | CPC 21502 | *Hibiscus sabdarifa* | Senegal | KF951924 | KF951991 | KF951797 | KF952159 | KF952239 |
|  | WAC 2767 | *Arachis hypogaea* | Australia | MW592083 | MW592164 | MW591657 | MW592241 | MW592327 |
| *M. tecta* | BRIP 70720 | *Sorghum bicolor* | Australia | MW592110 | MW592136 | MW591627 | MW592269 | MW592298 |
|  | **BRIP 70781** | *S. bicolor* | Australia | MW592058 | MW592138 | MW591684 | MW592271 | MW592300 |
|  | BRIP 70729 | *Vigna radiata* | Australia | MW592108 | MW592190 | MW591629 | MW592267 | MW592354 |

^a^Acronyms of culture collections: BRIP: Queensland Plant Pathology Herbarium, Brisbane, Qld, Australia; CBS: Culture Collection of the Westerdijk Fungal Biodiversity Institute, Utrecht, the Netherlands; CMM: Coleção de Culturas de Fungos Fitopatogênicos Prof. Maria Menezes; CPC: Culture collection of Pedro Crous, housed at CBS; USQ: fungal collection of Centre for Crop Health, University of Southern Queensland, Toowoomba, Queensland, Australia; WAC: Western Australian Plant Pathology Reference Culture Collection, Perth, WA, Australia. Ex-type isolates are indicated in bold.
